# The Use of Tele-Music Interventions in Supportive Cancer Care: A Systematic Review

**DOI:** 10.3390/brainsci15121266

**Published:** 2025-11-25

**Authors:** Lore Mertens, Laura Tack, Tom Boterberg, Jörg Fachner, Leonardo Muller-Rodriguez, Marte Vandeweyer, Sofie Demasure, Marianne Hanssens, Tine Loyson, Laurence Goethals, Isabelle Kindts, Hannelore Denys, Patricia Schofield, Mohammad Najlah, Philip R. Debruyne

**Affiliations:** 1 Department of Medical Oncology, OECI-Designated Kortrijk Cancer Centre, General Hospital Groeninge, 8500 Kortrijk, Belgium; lore.mertens@azgroeninge.be (L.M.);; 2 Department of Human Structure and Repair, Ghent University, 9000 Ghent, Belgium; 3 Department of Radiotherapy, Ghent University Hospital, 9000 Ghent, Belgium; 4 Cambridge Institute for Music Therapy Research (CIMTR), Anglia Ruskin University, Cambridge CB1 1PT, UK; 5 Department of Radiotherapy, OECI-Designated Kortrijk Cancer Centre, General Hospital Groeninge, 8500 Kortrijk, Belgium; 6 Department of Medical Oncology, Ghent University Hospital, 9000 Ghent, Belgium; 7 School of Nursing and Midwifery, Faculty of Health, University of Plymouth, Plymouth PL4 8AA, UK; 8 Biomedical and Health Innovation Research Centre (BHIRC), School of Allied Health and Social Care, Faculty of Health, Medicine and Social Care, Anglia Ruskin University, Chelmsford CM1 1SQ, UK

**Keywords:** telehealth, music interventions, music therapy, supportive cancer care, complementary care, psychosocial functioning

## Abstract

**Objectives**: This systematic review seeks to provide an in-depth overview of current research on tele-music interventions in supportive cancer care and identifies key areas where further research is warranted. **Methods**: A comprehensive search was conducted across four electronic databases (Scopus, Embase, Web of Science, and PubMed) without any data restrictions and according to the PRISMA guidelines. The primary outcome measure was the effect of tele-music interventions on psychosocial functioning. **Results**: Of the 2.043 articles initially identified, nine studies met the inclusion criteria and were selected for qualitative analysis. Among the music interventions, considerable variation was observed regarding delivery format and techniques employed. Most interventions were delivered remotely through Zoom, and in all but one study, a music therapist was involved. Outcome measures addressed various psychosocial and physical symptoms, most frequently anxiety, for which findings were mixed: three studies reported significant reductions, whereas two others observed no or only limited improvement. **Conclusions**: The results suggest that tele-music interventions are effective in reducing a range of cancer-related symptoms, including stress, anxiety, depression, and pain. However, the heterogeneity in study designs and methodological limitations hampered direct comparison and overall effectiveness assessment. Additionally, digital technologies hold considerable potential for the accessible and cost-effective delivery of music interventions.

## 1. Introduction

Despite significant advances in detection and treatment, cancer remains one of the leading causes of morbidity and mortality worldwide [1], and patients as well as survivors continue to experience substantial physical, psychological, and social burdens [2]. In addition to the more common physical side effects such as nausea, fatigue, and pain [3,4], an increasing number of patients and survivors are reporting symptoms of depression [5], anxiety, distress, fear of disease progression or recurrence [6], sleep difficulties, cognitive complaints [7,8,9], and mood disturbance. These symptoms transcend the treatment phase and impact overall well-being and quality of life.

In response to the multifaceted impact, there has been growing interest in and demand for integrative interventions alongside standard pharmacological cancer treatments [2,10,11]. Physical exercise, psychological counseling, nutritional advice, and mind–body techniques, such as yoga, acupuncture, mindfulness meditation, and emotional freedom techniques (EFT), are only a few examples of evidence-based complementary interventions that are gaining popularity [6,9,12,13,14,15].

Another complementary approach that has received considerable recognition in supportive cancer care is the use of music-based interventions. Originally applied mainly in mental health, geriatric, and pediatric care, music is increasingly being integrated into oncology settings, where it has been shown to enhance both psychological and physical well-being [16,17,18]. Additionally, music therapy is acknowledged in the NCCN Guidelines for Distress Management as a valuable complementary therapy for patients experiencing depressive symptoms, anxiety, or general distress [19].

When talking about music interventions, it is essential to delineate distinctly between music therapy, music listening (including music medicine), and other music-based approaches [20,21,22]. Firstly, music therapy requires the involvement of a board-certified music therapist and is defined as the psychotherapeutic use of music to address physical, emotional, cognitive, communicative, and social needs within a therapeutic relationship [23]. It can encompass both active musical activities, such as instrument playing, singing, and songwriting, and more receptive techniques, such as music-assisted relaxation or Guided Imagery and Music (GIM) [24]. Alternatively, the music listening approach does not require a therapeutic relationship between patient and therapist, but employs music listening to pursue health-related goals [20]. This approach comprises both individualized music listening and music medicine. The former uses carefully compiled playlists, consisting of music tracks that are emotionally relevant for the patient. The latter involves the use of music tracks chosen for their structural properties and aligned with a specific therapeutic objective. The ‘Music as Medicine’ research group at Erasmus Medical Center (Rotterdam, The Netherlands), for example, has been researching the therapeutic effect of music pre-, during, and post-surgery for many years [25]. In cancer care settings, purposeful music listening can be highly valuable in alleviating common physical and psychosocial complaints experienced by patients [16,26]. Lastly, generic music-based approaches are non-specific interventions that utilize music to improve well-being without necessitating a predefined therapeutic framework or the involvement of a music therapist [22].

Despite evidence supporting their effectiveness across multiple contexts and outcome measures, music interventions have not yet been integrated into standard cancer care protocols, which limits both their accessibility and reimbursement [16]. In addition, rising demands on healthcare systems and the associated costs underscore the need for interventions that are both accessible and cost-effective [27,28]. The adoption of digital and innovative technologies for delivering supportive cancer care has significantly increased [29] and may represent a viable strategy to address these challenges. Thus, the remote delivery of music interventions via telehealth may enhance patient access, reduce logistical and financial barriers [30], and support broader integration into standard supportive cancer care protocols.

Various digital technologies are currently available that enable the remote delivery of music interventions. However, clear and consistent terminology is essential in this emerging field: (a) eHealth (i.e., electronic Health) is considered an umbrella term, and can be defined as the broad use of electronic systems to support healthcare services, (b) telehealth refers specifically to the remote delivery of healthcare by means of telecommunications technology, (c) mHealth (i.e., mobile Health) uses mobile devices (smartphone, tablet, smartwatch) to facilitate continuous healthcare through monitoring and communicating with patients.

To this date, little has been written about the remote delivery of music interventions for this specific population. This systematic review, therefore, aims to (1) synthesize the existing, albeit limited, literature on tele-music interventions for cancer patients, (2) explore the effectiveness, feasibility, and acceptability of these interventions, and (3) inform clinical practice and highlight directions for future research in this field.

## 2. Materials and Methods

This review was conducted in accordance with the Preferred Reporting Items for Systematic Review and Meta-Analyses (PRISMA) Statement [31] (Table S1) and registered in the International Prospective Register of Systematic Reviews (PROSPERO), with the study protocol publicly accessible (ID: CRD420251068775).

### 2.1. Search Strategy

A sensitive and systematic literature search was conducted between March 2025 and June 2025. Databases included Scopus, Web of Science, Embase, and PubMed. The PubMed syntax can be found in Appendix A. To ensure consistent terminology and search effectiveness, this search string was adapted to the other databases using Polyglot Search Translator [32]. Only articles in Dutch or English were considered eligible. No search data restrictions were applied.

### 2.2. Inclusion and Exclusion Criteria

To be included in the review, studies had to (a) contain original research data, (b) deal with adults with a past or current cancer diagnosis of any type and at any stage, (c) involve remotely delivered music interventions in any form (active or receptive; through teleconferencing, mobile applications or web-based platforms; using personalized playlists or internet-based programs combining music therapy with mindfulness; among others), and (d) evaluate feasibility or the impact on psychosocial functioning and/or symptom burden and/or quality of life. Studies in which music interventions were offered completely in-person or that required the physical presence of a music therapist or healthcare professional were excluded. Studies reporting on interventions where music was only a minor, irrelevant part (e.g., background music during meditations) were also excluded.

Since the field of tele-music interventions within supportive cancer care is still largely unexplored and high-quality randomized trials are expected to be scarce, a broader inclusion approach in terms of study design was adopted. Accordingly, studies with lower methodological rigor, such as case series or conference abstracts, were also considered for inclusion in this review. Study protocols, commentaries, and other publications lacking original empirical data were not considered eligible.

### 2.3. Study Selection and Data Extraction

Articles were selected using a two-step screening process, facilitated by the web-based automated screening tool Rayyan [33]. Two authors (L.M., L.T.) independently screened all potentially relevant full texts. Any discrepancies were resolved through discussion.

Relevant data were extracted from all included studies using a standardized data sheet. This included study design, population characteristics, intervention type, outcomes assessed, and key findings relevant to the review question. Data extraction was performed by one reviewer (L.M.).

Given the limited number of randomized controlled trials (RCTs) and the qualitative nature of several included studies, a narrative synthesis approach was used. Findings were summarized thematically, structured around the type of intervention, target population, and reported outcomes. Where possible, similarities and differences across study designs and contexts were highlighted.

### 2.4. Risk of Bias Assessment

Risk of bias was assessed for all included studies using design-specific assessment tools: the Cochrane Risk of Bias 2 tool for RCTs [34], the Risk Of Bias In Non-randomized Studies—of Interventions (ROBINS-I) for non-randomized trials [35], and the corresponding Joanna Briggs Institute (JBI) Critical Appraisal Checklists for case series [36] and qualitative studies [37].

## 3. Results

**Figure 1:**
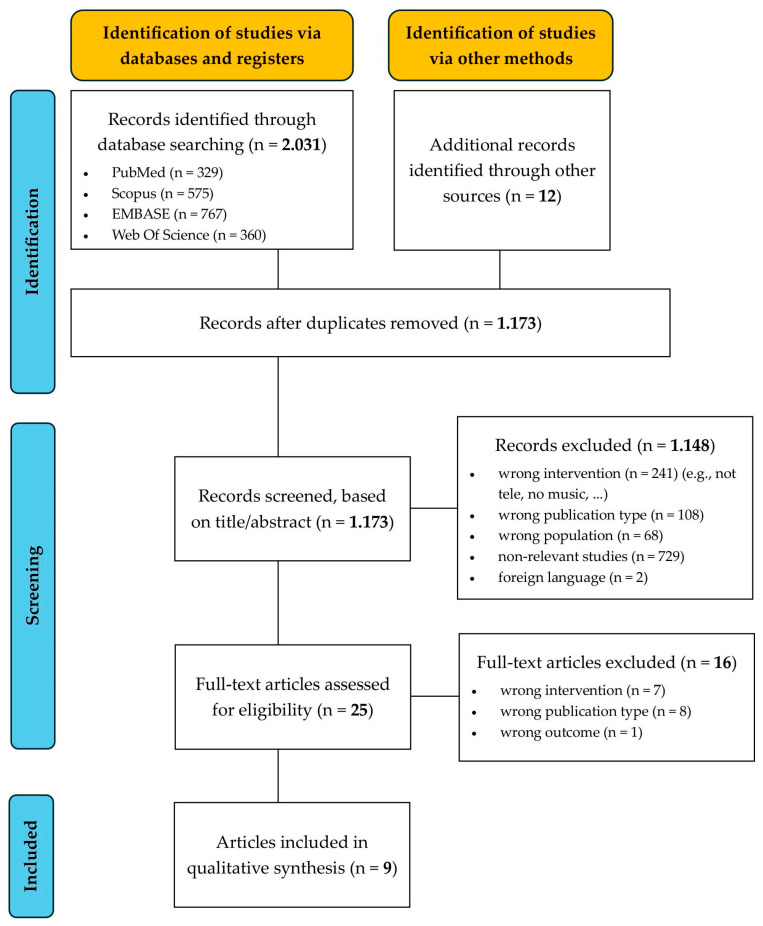
PRISMA 2020 flow diagram for systematic reviews.

As shown in the PRISMA flow diagram (Figure 1), the initial database search yielded 2.031 publications, of which 1.173 remained after removal of duplicates. In addition, 12 articles were identified through other sources, primarily by screening the reference lists of recent reviews. After a first-stage screening on title and abstract, 1.148 records were excluded for the following reasons: wrong intervention (*n* = 241), wrong publication type (*n* = 108), foreign language (*n* = 2), not relevant (*n* = 729). Finally, full-text screening of the remaining 25 articles resulted in the inclusion of nine studies that met the eligibility criteria. Selected studies, key features, and the main results can be found in Table 1.

### 3.1. General Information

Only three of the included studies were RCTs [38,39,40]. Of these, one is currently available only as a conference abstract and does not yet have a full-text publication (under review [40]). This reflects the scarcity of robust, high-quality research in the field so far.

Three other publications were included, each reporting on distinct aspects of the same underlying mixed-methods trial [41,42,43]. Knoerl et al. (2022) [41] presented the main quantitative outcomes; Phillips et al. (2023) [42] reported on the qualitative findings; and Knoerl et al. (2023) [43] performed a secondary analysis on the original dataset. To avoid giving undue weight to this study in the synthesis, these three publications [41,42,43] were treated as multiple reports of a single study. Furthermore, the selection for this review also included a case series [44], a qualitative study [45], and a quasi-experimental study [46].

Most studies were conducted in the USA [40,41,42,43,44,45], two in Turkey [38,46], and one in Israel [39]. Sample sizes of the nine selected studies ranged from 2 to 300. All studies involved patients of both genders, with a predominance of female participants. Study populations were heterogeneous with respect to cancer type, as participants were recruited across a range of malignancies, with breast cancer being the most prevalent diagnosis reported. However, one study exclusively investigated patients with acute myeloid leukemia who received allogeneic hematopoietic stem cell transplantation (allo-HSCT) [45].

### 3.2. Delivery Format

While some studies were originally designed for digital delivery, others adopted this format in response to the constraints imposed by the COVID-19 pandemic. Consequently, the mode of delivery varied: one study utilized a purpose-built digital platform [45], another provided pre-recorded music via MP3 players [46], and the seven remaining studies delivered the digital interventions synchronously through Zoom [38,39,40,41,42,43,44].

### 3.3. Music Intervention Characteristics

A variety of techniques were used across the studies, with music often being integrated with mindfulness practices. Examples of this are music-assisted (progressive muscle) relaxation [41,44,45], breathing to music [38,40,45,47], or performing a music-supported body scan [45]. Other techniques included psychoeducation [38,41], listening to music [39,46], playlist creation [40], songwriting [40,44], and vocal improvisation [45]. Regarding music listening, two unique—the three articles reporting on the same mixed-methods trial [41,42,43] count as one study here—studies implemented live music performances [41,42,43,44], whereas the others relied on pre-recorded music [38,39,40,45,46]. In all but one study [46], a board-certified music therapist was involved. Across the selected studies, three of them investigated group-based interventions [38,39,40], three other studies delivered individual one-on-one sessions [41,42,43,45,46], and one study combined both approaches [44]. The number of intervention sessions ranged from 1 to 8, each lasting 30 to 60 min, with intervention periods spanning from a single day [39] to over three months [45].

### 3.4. Outcome Measures

Generally, a variety of outcome measures were employed to evaluate the effects of music interventions: five studies relied exclusively on self-report questionnaires; two studies employed a combination of semi-structured interview questions, observational methods, and self-report questionnaires, and one study conducted only semi-structured interviews. Regarding the study by Liou and colleagues, the study protocol that was published in 2023 [48] mentions the use of both self-report questionnaires and semi-structured interviews, assessing anxiety and a range of secondary outcomes (depression, fatigue, pain, insomnia, etc.). However, in the meeting abstract [40] included in this review, no details are provided regarding the results of the other outcome measures. Therefore, only the results relating to anxiety will be considered here.

All but one study focused on the impact of the intervention on anxiety (reduction). However, evidence is mixed, with three studies reporting a significant decrease in anxiety scores [40,44,46] and two studies observing no or only limited improvement [39,41].

Other common trends were the assessment of distress, depression, stress and pain, with many studies utilizing validated scales such as the Hospital Anxiety and Depression Scale (HADS) [49], State Trait Anxiety Inventory (STAI) [50], Perceived Stress Scale (PSS) [51], Edmonton Symptom Assessment System (ESAS) [52], and the Beck Depression Inventory (BDI) [53]. Additionally, several studies included more general well-being measures (e.g., Psychological Well-being Scale [54], Patient-Reported Outcomes Measurement Information System (PROMIS) measures [55], General Comfort Questionnaire [56]).

In addition, four studies incorporated questionnaires (i.e., the Usefulness, Satisfaction and Ease of Use (USE) questionnaire [57], the Acceptability E-Scale [58]) or qualitative interviews to better understand patient experiences and assess the feasibility or acceptability of an intervention [41,42,44,45].

### 3.5. Risk of Bias Assessment

As mentioned, all selected articles were subjected to a risk of bias assessment. Design-specific tools were used, with the Cochrane Risk of Bias 2 (RoB) tool for RCTs [34], the ROBINS-I for non-randomized trials [35], and the corresponding JBI Critical Appraisal Checklists for case series [36] and qualitative studies [37]. Results can be found in Table 2.

**Table 1:** Characteristics of included studies in alphabetical order.

Study	Country + Year	Design	Subjects	Intervention + Delivery Format	Outcome Measures	Key Findings
Bilgiç and Acaroğlu [46]	Turkey (2016)	Quasi-experimental study	Adult patients receiving chemotherapy (*n* = 70)	Music listening, during chemotherapy and the week after (at home)	Edmonton Symptom Assessment System (ESAS), General Comfort Questionnaire (GCQ), patient observation form	Significant between-group mean difference in chemotherapy symptoms (pain, exhaustion, nausea, anxiety, lethargy, lack of appetite, not feeling well). Significant improvements in total general comfort: physical, sociocultural, and psychospiritual comfort.
Fleszar-Pavlovic et al. [45]	USA (2025)	Qualitative study (focus groups, usability, and field testing)	Patients post allo-HSCT for myelodysplastic syndrome or leukemia (≥3 months in remission) (*n* = 11)	8 modules eHealth-delivered mindfulness-based music therapy (eMBMT) intervention (8 modules)	(a) Interview questions regarding challenges, usefulness, and preferences. (b) Field testing using the think-aloud method (c) Usefulness, Satisfaction, and Ease of Use (USE) questionnaire	Positive evaluations for usefulness, ease of use, and satisfaction with the eMBMT platform 8. Identified areas for improvement (content representativeness, reduced text, enhanced guidance, diverse music options).
Folsom et al. [44]	USA (2021)	Case series	Cases on patients receiving care within an integrative oncology setting (*n* = 2)	Outpatient telehealth group music therapy, in- and outpatient individual telehealth music therapy (iPad and Zoom application)	Symptom burden (Edmonton Symptom Assessment Scale), patient feedback interview (open-ended questions regarding patients’ experience)	Reduced anxiety, increased coping skills, enhanced social support, improved mood, and greater convenience
Knoerl et al. [41] *	USA (2022)	Single-arm pre/post-test intervention study	Adolescents and young adults receiving chemotherapy (*n* = 37)	Up to four individual mindfulness-based music therapy sessions (in-person or via Zoom) over twelve weeks	Anxiety (Patient-Reported Outcomes Measurement Information System Anxiety 4a), stress (Perceived Stress Scale), acceptability (Acceptability E-Scale)	Significant improvements in perceived stress, non-significant changes in anxiety. Highly rated satisfaction and acceptability
Knoerl et al. [43] *	USA (2023)	Secondary analysis of a single-arm pre-/post-test intervention study	Adolescents and young adults receiving chemotherapy (*n* = 31)	Up to four individual mindfulness-based music therapy sessions (in-person or via Zoom) over twelve weeks	Perceived Stress Scale, Post-Traumatic Growth Inventory Short Form, Patient-Reporting Outcome Measurement Information System	Potential influencing factors associated with anxiety improvement: higher baseline physical functioning, anxiety, fatigue, sleep disturbance, female sex, or virtual intervention delivery
Liou et al. [40]	USA (2025)	RCT (reported in a meeting abstract)	Cancer survivors (*n* = 300)	7-weekly telehealth-based music therapy (MT) vs. cognitive behavioral therapy (CBT) via Zoom	Primary: Hospital Anxiety and Depression Scale (HADS) Secondary: depression, fatigue, insomnia, pain, cognitive function, quality of life	MT was non-inferior to CBT for short- and long-term anxiety reduction. Both treatments produced clinically meaningful, durable anxiety reduction.
Phillips et al. [42] *	USA (2023)	Qualitative study	Young adults (20–39 years old) receiving chemotherapy (*n* = 16)	Up to four individual mindfulness-based music therapy sessions (in-person or via Zoom) over twelve weeks	Semi-structured interviews on (1) reasons for participating, (2) prior music/mindfulness experience, (3) usual coping strategies, (4) experiences with participation, (5) use of strategies after study completion, (6) suggestions for improving the intervention	(1) Privacy issues attending virtual sessions, (2) sense of relaxation, (3) sense of connection to the music (in-person) and connection to mindfulness (virtual), (4) synergistic feeling practicing music and mindfulness together (virtual), (5) in-person delivery preferred to virtual delivery
Rabinowitch et al. [39]	Israel (2023)	RCT	Patients with current cancer diagnoses, either under or in between treatments (*n* = 30)	Online group music listening intervention (Balance-Space) vs. online group meditation intervention	Primary: NCCN Distress Thermometer; State Trait Anxiety Inventory (STAI-S); Visual Analog Scale (VAS for pain) Qualitative analysis of post-session open discussions	Significant reduction in perceived pain levels following the music intervention. No significant differences in distress or anxiety levels
Yildirim et al. [38]	Turkey (2024)	RCT	Patients with current cancer diagnoses, all receiving treatment at the University Hospital in Istanbul (*n* = 120)	10-day online mindfulness-based stress reduction program combined with music therapy. Control group received standard care	Primary: State Trait Anxiety Inventory-State (STAI-S); Psychological Well-Being Scale (PWBS); Beck Depression Inventory (BDI)	Significant reduction in stress and depression, and higher psychological well-being in the intervention group

* Articles reporting on the same (virtual) mindfulness-based music intervention with the same (subset of) participants, but a different research focus and design.

**Table 2:** Risk of bias in the included studies.

Cochrane Risk of Bias (RoB) 2											Risk of Bias in Non-Randomized Studies—Interventions (ROBINS-I)										
	Bias due to randomization		Deviations from intended intervention		Missing outcome data	Outcome measurement		Selection of reported results	Overall			Classification of interventions	Deviations from intended intervention		Missing outcome data	Outcome measurement		Selection of reported results	Confounding	Selection of participants for the study	Overall
Yildirim et al. (2024) [38]	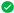		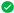		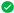	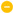		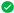	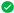		Bilgiç and Acaroğlu (2017) [46]	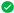	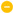		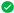	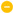		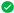	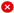	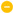	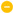
Rabinowitch et al. (2023) [39]	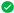		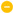		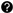	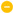		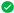	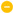		Knoerl et al. (2022) [41]	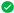	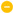		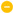	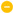		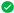	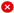	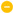	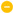
Liou et al. (2025) [40]	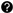		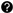		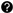	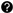		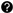	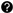		Knoerl et al. (2023) [43]	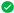	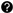		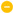	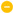		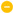	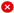	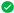	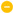
**JBI Critical Appraisal Checklist for Case Series**											**JBI Critical Appraisal Checklist for Qualitative Research**										
	Clear criteria for inclusion	Condition measurement	Valid identification methods	Consecutive inclusion	Complete inclusion	Reporting of demographics	Reporting of clinical information	Reporting of outcome results	Reporting of the presenting site demographic info	Appropriate statistical analysis		Philosophy ≅ methodology	Methodology ≅ research questions	Methodology ≅ data collection	Methodology ≅ data representation and analysis	Methodology ≅ interpretation	Researched is culturally/theoretically situated	Addressing the influence of the researcher	Representation of participants	Ethical approval	Relationship of conclusion to analysis
Folsom et al. (2021) [44]	No	Yes	Yes	No	No	Yes	Yes	Yes	Yes	NA	Phillips et al. (2023) [42]	No	Yes	Yes	Yes	Yes	No	No	Yes	Yes	Yes
											Fleszar-Pavlovic et al. (2025) [45]	No	Yes	Yes	Yes	Yes	No	No	Yes	Yes	Yes

Judgment: 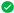: Low risk; 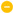: Moderate risk; 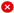: High risk; 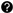: Unclear; NA: Not applicable.

## 4. Discussion

The primary aim of this review was to map and synthesize existing research on tele-music interventions in supportive cancer care. Despite the limited amount of high-quality evidence available, the findings suggest that tele-music interventions may contribute to reducing psychosocial burdens and improving patients’ quality of life. Given the ongoing digitalization of healthcare and society, tele-music interventions are of particular interest, as they have the potential to increase accessibility to complementary care for a broader patient population.

### 4.1. Key Findings

#### 4.1.1. Methods and Components of Tele-Music Interventions

Considerable variation was found in the music interventions studied. In some cases, the content of the intervention was shaped by the chosen mode of virtual delivery. For example, in the study by Phillips and colleagues [42], the transition to an online format hindered active music-making, because patients did not have access to musical instruments, and sound delays made it practically impossible to play with others. Thus, in digital formats, mainly receptive music activities (i.e., music listening) were offered. However, this does not necessarily constitute a limitation, since research has shown that both active and receptive music interventions can enhance psychological well-being [59]. Moreover, patients with no prior experience of music interventions often demonstrate a stronger initial preference for receptive modes of engagement [60,61]. Offering music in such a low-threshold format also allows patients to benefit from the experience without facing the barrier of a (perceived) lack of musical competence [62].

In all studies except one [46], a board-certified music therapist was involved to deliver the music intervention. This underscores the central role of the music therapist in music-based care, but it also raises some important considerations. First, reliance on board-certified music therapists increases the implementation costs, thereby hampering large-scale adoption. Second, their presence might introduce therapist effects (e.g., individual attention, relational factors) that are rarely accounted for in study designs, making it difficult to determine the specific contribution of music itself. Finally, the predominance of provider-led approaches highlights the lack of tele-music interventions that patients can access independently and on demand (i.e., self-managed). This enduring reliance on professional support limits patient empowerment and prolongs dependency on healthcare providers, even beyond active treatment. Further research is needed to clarify the unique contribution of the music therapist in remote interventions, possibly through non-inferiority trials comparing therapist-led interventions with nurse-led interventions, and more autonomous, client-driven approaches.

#### 4.1.2. Psychosocial Outcomes of Music Interventions

Music-based interventions have repeatedly shown to reduce a range of cancer-related symptoms, including stress, anxiety, depression, pain, and to improve overall well-being and comfort. These effects were observed in diverse cancer populations (young people and adults, with different types of cancer and at different stages of their treatment). Notably, multiple studies document effect sizes that meet or exceed minimal clinically important differences, indicating benefits that are not just statistically significant but also clinically meaningful to patients [38,40].

In nearly all studies, the potential effect of music on anxiety levels was explored. However, findings have been mixed, with three studies reporting significant reductions in anxiety [38,40,46] and two studies showing non-significant, limited, or even no improvements [39,41]. Knoerl and colleagues [43] extended this line of inquiry by investigating factors that may moderate the effect. Their findings indicate that higher baseline levels of anxiety and physical functioning, along with sleep disturbances, fatigue, and female sex, were associated with greater reductions in anxiety following a music intervention. Interestingly, participants who engaged in online sessions demonstrated greater improvements than those attending in-person sessions. While further research is required to confirm these results, this suggests that the mode of delivery, among other factors, may impact the extent to which individuals benefit from music interventions.

Additionally, more research is needed into the mechanisms that may underlie the effects of tele-music interventions [39]. Several mechanisms known from in-person music therapy are likely to operate similarly in remote formats, including modulation of different physiological responses (blood pressure, heart rate, endocrine system (cortisol, dopamine, oxytocin)), affective and cognitive aspects of pain regulation, and emotion-regulation processes [63]. Psychological mechanisms such as distraction, a sense of empowerment, and perceived social connection may also be preserved in synchronous online (group) sessions [39].

#### 4.1.3. Acceptability and Feasibility

Overall, most interventions were well-received in terms of feasibility and satisfaction. Participants recognized the advantages of delivering music interventions remotely: it enhances accessibility and may help maintain continuity of care [45] by providing greater scheduling flexibility [41] and reducing mobility barriers. Despite these strengths, participants in two studies expressed a preference for in-person delivery over virtual formats [42,45]. In the qualitative study by Phillips and colleagues [42], for example, all participants in the virtual group stated they would have joined in-person sessions, whereas only three of seven in-person participants would have participated virtually. Interestingly, however, recruitment and attendance increased after transitioning to online sessions, and completion rates were higher in the virtual condition than in the in-person group [41,64]. Taken together, these findings suggest a distinction between what is preferred and what is feasible in the context of tele-music interventions. This tension may partly reflect the technological and logistical barriers that persist, despite significant advances in telehealth over the past decade. Patients described stress related to coordinating virtual sessions alongside hospital visits and leisure time, difficulty securing sufficient privacy at home, inadequate understanding of the digital tools, and, in some cases, symptoms of screen fatigue [42,45].

These findings underscore the importance of carefully selecting delivery formats and implementing user-friendly tools with low technical burden to empower patients in their recovery, both during and after treatment. An excellent example of this is SmartManage, a web-based telehealth platform that centralizes all intervention content, supports real-time session engagement, and enables independent practice across multiple devices [47].

### 4.2. Strengths and Limitations

In this review, a systematic search across multiple databases was conducted to ensure comprehensiveness and reproducibility, guided by clear inclusion and exclusion criteria. However, the inclusion of lower-level evidence studies introduces some limitations that warrant careful consideration.

First, the overall quality of evidence remains low, dominated by small pilot studies, case series, and non-randomized designs, which reduces generalizability and heightens concerns about bias and confounding. Second, the substantial heterogeneity in study designs and outcome measures impeded direct comparisons and precluded meta-analysis. Some studies used active controls (e.g., meditation), whereas others opted for passive controls (i.e., standard care) or no control group. Third, the lack of standardized reporting and delivery of interventions undermined interpretability and replicability, highlighting the need for adoption of established reporting guidelines, such as those proposed by Robb and colleagues [65]. Lastly, the risk of bias assessments was conducted by a single reviewer, which may have introduced subjectivity and limited the robustness of the evaluation.

### 4.3. Clinical Implications and Future Directions

This review contributes to the existing, albeit limited, knowledge base and hopes to inform clinicians, researchers, and policymakers about the current state of the field, highlighting both best practices and gaps where further investigation is needed.

At the same time, the observed heterogeneity across interventions, patient groups, and outcome measures warrants careful consideration when translating these findings into clinical practice. This variability makes it difficult to identify which tele-music formats are most effective, yet it also suggests that different approaches may be better suited to different clinical needs. Future research should therefore clarify which intervention components work best for whom and under which circumstances.

Much is to be expected from future developments in the field of tele-music interventions. The recently published protocols and meeting abstracts of research projects currently being conducted bear witness to this [40,47,66,67]. In the coming years, technological advances such as artificial intelligence (AI) [67,68], virtual reality (VR) [69], and wearable devices [70] are expected to become increasingly integrated into tele-music interventions.

Moreover, integrating evidence from both music therapy and music medicine studies will be essential to support the implementation of tele-music interventions in cancer care. Doing so will help align clinical practice with current standards for evidence-based music interventions. Music is a complex multidimensional stimulus whose psychoneurobiological effects depend on the characteristics of the music being listened to or performed [71,72]. Research in this field underscores the importance of personalizing music and adapting its features to the intended emotional or arousal goal, as well as to the listener’s context [73,74].

Additionally, considered the gold standard for evaluating interventions, RCTs are indispensable for proving the effectiveness of new techniques. Therefore, there is a need for more RCTS to further establish the potential of tele-music interventions in clinical practice.

## 5. Conclusions

To our knowledge, this is the first systematic review to focus specifically on the use of tele-music interventions in supportive cancer care. In an era where cancer care is increasingly focused on addressing both the physical and psychological dimensions, there is a growing interest in accessible and patient-centered complementary interventions. The findings suggest that music may help alleviate psychosocial burdens both during and after cancer treatment, and that virtual delivery represents a promising way to provide this intervention in a cost-effective and accessible manner. Future high-quality studies are essential to strengthen the evidence base and guide the integration of tele-music interventions into routine care.

## Supplementary Materials

The following supporting information can be downloaded at: https://www.mdpi.com/article/10.3390/brainsci15121266/s1, Table S1. PRISMA 2020 Checklist. Reference [75] is cited in the supplementary materials.

## Appendix A. PubMed Syntax

(((Oncology [Tiab] OR Oncolog* [Tiab] OR neoplasm* [Tiab] OR neoplasms [MeSH] OR Cancer* [Tiab] OR Tumor* [Tiab] OR Tumour* [Tiab] OR Carcinoma [Tiab] OR cancer care [Tiab]))
**AND**
((Music [MeSH] OR Music therapy [MeSH] OR music therap* [Tiab] OR music-based intervention* [Tiab] OR music based intervention* [Tiab] OR music medicine [Tiab] OR vocal therapy [Tiab] OR sing [Tiab] OR singing [Tiab] OR Singing [MeSH] OR song* [Tiab] OR vocal* [Tiab] OR melod* [Tiab] OR auditory stimulat* [Tiab] OR acoustic stimulation [MeSH] OR Music listening [Tiab] OR audio [Tiab] OR audio therap* [Tiab] OR audio-based [Tiab] OR sound [Tiab] OR sound therap* [Tiab] OR Receptive Music [Tiab] OR playlist* [Tiab]))
**AND**
((Telemedicine [MeSH] OR Mental health Teletherapy [MeSH] OR telecare [Tiab] OR tele-care [Tiab] OR tele care [Tiab] OR Mobile health [Tiab] OR mHealth [Tiab] OR Telehealth [Tiab] OR Teletherapy [Tiab] OR eHealth [Tiab] OR Remote [Tiab] OR digital* [Tiab] OR online [Tiab]))
**AND**
((Psychosocial Functioning [MeSH] OR Psychosocial [Tiab] OR Psychosocial treatment* [Tiab] OR psychological [Tiab] OR need* [Tiab] OR benefit* [Tiab] OR outcome* [Tiab] OR well-being [Tiab] OR wellbeing [Tiab] OR health-related outcome* [Tiab] OR Anxiety [Tiab] OR Depression [Tiab] OR Depressiv* [Tiab] OR Pain [Tiab] OR Fatigue [Tiab] OR Sleep [Tiab] OR Stress [Tiab])))
